# Transforming Health Sciences Library Spaces

**DOI:** 10.5195/jmla.2020.828

**Published:** 2020-01-01

**Authors:** Martha F. Earl

**Affiliations:** Preston Medical Library, Graduate School of Medicine, University of Tennessee, Knoxville, TN, mearl@utmck.edu

In the past twenty years, libraries have faced challenges of shifting technologies, fluctuating budgets, changing accreditation standards, and shrinking physical spaces. Despite dour warnings of the closing of physical libraries and the fading of the library profession, librarians have adapted services and repurposed library spaces to meet the needs of diverse clientele. Health sciences libraries serve hospitals, academic institutions, businesses, agencies, and the public. Those who manage those spaces have met the needs of new generations of users innovating to keep those users engaged.

Alanna Campbell, public services librarian at the Northern Ontario School of Medicine, addresses the changing landscapes in this collection of case studies. Campbell focuses on how health sciences libraries have realized the need that clientele have for transforming the library into a gathering space. The library as a community center includes study spaces, quiet areas, meeting rooms, makerspaces, copy services, information technology (IT) support, writing centers, cafes, and think tanks. None of these services is unique to libraries; however, the library that partners with other departments or presents the most appealing use of their own space can prevail as the heart of a campus or community.

Despite the diversity of health sciences libraries, the same issues surface: identifying and overhauling outdated and inflexible spaces, gathering data on how clientele use resources and asking them what they want, translating that feedback into satisfactory responses despite limited funding, removing the majority of the print collection and deciding on what to put in its place, maximizing relationships with stakeholders to update space, understanding the reality of going all digital, and managing use of materials that are not traditionally well suited to online access. The contributors to this volume discuss planning, budgeting, collecting and integrating user feedback, working with leadership and architects, and weathering the positive and negative aspects of change.

The book’s three sections are part I, “Library Spaces That Work for Users”; part II, “Working in Unique Spaces”; and part III, “Library Spaces Working with What They’ve Got,” and the ten chapters highlight key points. In part I, Roksandic and Erlinger detail the transformation of consumer health spaces at multiple sites associated with Mount Carmel Health Services and the renovation of the main Health Sciences Library as well. Lackey and colleagues describe the innovations at the University of Utah’s Eccles Health Sciences Library with Ithaka S+R. Most engaging in this chapter is their analysis of client engagement, sense of ambience, and other intangible assets of the library. At the University of Florida’s Marston Science Library, Minson and colleagues elaborate on the large budget renovation of an academic library to add new technologies, services, and user conveniences.

In part II, Blackwell at Chamberlain University addresses the challenges in transforming to an all-virtual library serving multiple campuses. Epstein illustrates how to demonstrate strong leadership in virtual spaces, examines the library website “as place,” and outlines the role of leaders. At Texas A&M, Carrigan and Burford describe how they updated their special collections area.

In part III, DeCaro and Butcheck of Cleveland Health Sciences Library discuss how the loss of 50% of their space at Case Western Reserve in 2014 ultimately resulted in more efficient use. Hoogland examines surviving tight budgets, adding value to spaces, and retooling the role of librarians, including needed professional development and education for both practitioners and students. To conclude the book, Campbell and Fink detail how they incrementally found money in their budget to support renovations without a line item for that purpose.

This book is well illustrated with black and white photographs, tables, and graphs, with a “List of Illustrations” included. An appendix presents the Marston Science Library survey. The bibliography constitutes a thorough review of twenty-first century literature, including association standards and recommendations. The index is cross-referenced and includes figures and tables. The final section, “About the Editor and Contributors,” introduces the reader to each contributor.

Although numerous books discuss renovation of library spaces related to user-centric and technologically driven changes, Campbell addresses the specific needs of health sciences libraries. Any health sciences library concerned with space reductions, wanting to change the value of physical spaces, or reviewing the literature on these topics can begin with this book. The MLA Books Panel chose well in promoting and supporting Campbell’s publication. This text can be employed by library and information science educators as well as practicing librarians.

This book is highly recommended for any health sciences library. Other academic or special libraries may also find it valuable, along with universities supporting schools of information science.

**Martha F. Earl, AHIP,** mearl@utmck.edu, Preston Medical Library, Graduate School of Medicine, University of Tennessee, Knoxville, TN

